# Notes and News

**Published:** 1901-11-23

**Authors:** 


### NOTES AND NEWS.

A VIRCHOW Institute is to be established in Moscow by the Russian Government.

MR. GEORGE COURTAULD has endowed a bed at the Royal Free Hospital in memory of his daughter, the late Miss Louise Courtauld.

THE committee for the abatement of street noises is obtaining a very formidable number of signatures to the petitions they are preparing to place before the authorities.

THE Lord Bishop of London last Saturday unveiled a window in the chapel of the Cancer Hospital, Fulham Road, erected in memory of the late Dr. William Marsden, who founded the charity in 1851.

NEW wards have recently been added to the Grimsby Union Infirmary. They are to accommodate male and female patients. A separate maternity department has been added, and a total increase of 82 beds has been effected. The exterior of the buildings corresponds with those already in use, and the cost of the extension has been £8,000.

DURING the past fortnight there has been a considerable increase in the number of fresh cases of small-pox. The Metropolitan Asylums Board is considering a proposal to erect temporary hospital buildings to relieve the strain put upon the present accommodation. Large numbers of convalescents are now being removed to Gore Farm Hospital at Darenth.

MR. ALFRED JONES, the originator of the Liverpool School of Tropical Medicine, has been created a Knight Commander of the Order of St. Michael and St. George. In connection with the school, and largely owing to the initiative of Sir Alfred Jones, a hall of residence for students has been arranged in Liverpool, within easy residence of the Medical School and the Royal Southern Hospital. The cost of residence is wonderfully moderate, and it is hoped to attract students from the tropics, the hall being open to all races and creeds.

LADY SELFORD has laid the foundation stone of the extension of the General Hospital at Kettering. This addition includes a children's ward.

THE London School of Tropical Medicine numbers 25 new students for the present term, amongst them being officers of the Colonial Service and missionaries. The school has also several foreigners on its students' roll.

THE Great Northern Central Hospital has become possessed of a sum of £3,000, which has for some time been in dispute owing to the confused description given of the institution in the will of Miss Deleglise, of Canonbury, who bequeathed the money.

GLASGOW is naturally doing its best to remove the stigma of being a plague-infected city. The Lord Provost, in order to inspire confidence, has agreed to dine publicly in the Glasgow Central Hotel, where the cases originated, and to sleep there on the same evening.

THE Borough of St. Pancras, for the purpose of notification, has appointed six medical referees to make clinical examinations in doubtful or suspicious cases to be called in by the medical attendant of patients. Chicken-pox has now been declared a notifiable disease in the Borough.

IN answer to the special appeal of the committee organising the Coronation gift in connection with the Prince of Wales's Hospital Fund for London, the following amounts have been received at the Bank of England:—Messrs. Frank L. H. Collins and Son, £10 10s.; Miss S. Aste, £5; Messrs. A. and J. Main and Co., Limited, £5; A. B. Y., £3 3s.; Mr. F. P. Wells, £2 2s.; Mr. A. S. Bulkley, £2; Rev. F. Dunsby (collection), £1 12s. 6d.; Mr. F. H. Brown, £1 1s.; Miss Mary E. Fryer, £1 1s.; Mrs. Mary Randles, £1 1s.; Messrs. W. P. Thompson and Co., £1 1s.

THE following elections to the Army Medical Services Advisory Board have been made:—Chairman.—The Director-General Army Medical Service, Surgeon-General William Taylor, C.B., A.M.S., M.D., C.M. Vice-Chairman.—The Deputy-Director-General, Surgeon-General (temporary) Alfred Henry Keogh, C.B., A.M.S., M.D. Members.—Officer R.A.M.C. (Expert in Sanitation), Major Wm. Grant Macpherson, R.A.M.C., M.A., M.B., C.M., D.Ph. Camb.; Officer R.A.M.C. (Expert in Tropical Diseases), Lieutenant-Colonel David Bruce, R.A.M.C., M.D., C.M. Civilian Members.—Dr. Charles Bent Ball (Ireland), M.D., F.R.C.S. (Ireland), F.R.C.S. (England); Alfred Downing Fripp, Esq., C.B., C.V.O., F.R.C.S., etc.; Dr. Jas. Galloway (Scotland); M.A., M.D., F.R.C.S., F.R.C.P.; Dr. Edwin Cooper Perry, M.A., M.D., F.R.C.P.; Sir Frederick Treves, C.B., K.C.V.O., F.R.C.S. Representative of the War Office.—Colonel W. A. Dunne, C.B., Assistant Quartermaster-General. The permanent constitution of the Board will differ from the above in having only four civil members instead of five. The larger number is required at the outset in consequence of the heavy initial work necessitating a larger number of meetings than will be necessary later.

